# A tertiary care study on the effectiveness of moderate weight loss on heart rate variability frequency-domain components in obese patients at high cardiovascular risk

**DOI:** 10.31744/einstein_journal/2026AO1470

**Published:** 2026-01-02

**Authors:** Priscila Moreira, Cristiane Kovacs Amaral, Dalmo Antônio Ribeiro Moreira, Maria do Carmo Franco

**Affiliations:** 1 Universidade Federal de São Paulo Program of Translational Medicine Faculdade de Medicina São Paulo SP Brazil Program of Translational Medicine, Faculdade de Medicina, Universidade Federal de São Paulo, São Paulo, SP, Brazil.; 2 Instituto Dante Pazzanese de Cardiologia São Paulo SP Brazil Instituto Dante Pazzanese de Cardiologia, São Paulo, SP, Brazil.; 3 Universidade Federal de São Paulo Faculdade de Medicina Physiology Department São Paulo SP Brazil Physiology Department, Faculdade de Medicina, Universidade Federal de São Paulo, São Paulo, SP, Brazil.

**Keywords:** Heart disease risk factors, Diet therapy, Diet, healthy, Weight loss, Autonomic nervous system, Obesity

## Abstract

**Objective:**

Cardiovascular diseases remain the leading cause of mortality, and obesity is a significant risk factor. This study aimed to investigate the impact of a structured nutritional intervention on weight loss, heart rate variability, and biochemical profiles in obese patients with high cardiovascular risk.

**Methods:**

This was a non-randomized, uncontrolled study on the effectiveness of an institutional nutritional program. Heart rate variability measurements were obtained at baseline and after the nutritional program using a six-channel digital electrocardiograph.

**Results:**

This study included 24 patients with obesity (body mass index >30kg/m²), aged 42-80 years, who underwent a 3-month nutritional program. Significant reductions in body weight (p=0.043), body mass index (p=0.042), and waist circumference (p=0.031) were observed. Biochemical profiles improved, with decreased glycated hemoglobin (p=0.015), total cholesterol (p=0.001), LDLc (p=0.047), and triglycerides (p=0.021), while HDLc remained unchanged. Heart rate variability analysis revealed significant post-nutritional program decreases in low-frequency (p<0.001) and low-frequency/high-frequency ratios (p<0.001), and an increase in high-frequency (p<0.001), suggesting improved autonomic balance. No significant changes were observed in the heart rate, SDNN, pNN50, rMSSD, or triangular index.

**Conclusion:**

These findings suggest that moderate weight loss can positively influence autonomic function and several cardiovascular risk factors in obese patients with high cardiovascular risk. The nutritional program could be an important adjunctive strategy in the management of patients treated at tertiary care hospitals, offering a promising avenue for cardiovascular risk factor management and building a supportive environment for healthy eating habits.

## Highlights

■The institutional nutritional program in tertiary care was effective in promoting moderate weight loss.

■Obese patients with high cardiovascular risk undergoing multi-drug therapy showed significant improvement in anthropometric, biochemical, and heart rate variability components.

■Heart rate variability analysis revealed enhanced autonomic balance, increased parasympathetic activity, and reduced sympathetic activity after weight loss.

■Moderate weight loss presents a promising adjunctive strategy for managing cardiovascular risk in obese patients undergoing multi-drug therapy.

## Summary

Moderate weight loss achieved through a 3-month nutritional program improved the metabolic profile and autonomic function in obese patients at high cardiovascular risk, as evidenced by favorable changes in heart rate variability.

## Graphical abstract

**Figure:**
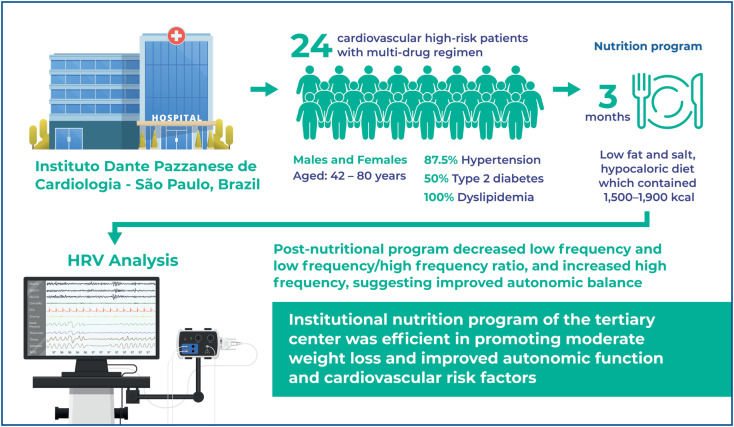

## INTRODUCTION

Cardiovascular diseases (CVDs) remain a major cause of mortality and morbidity worldwide.^(1)^ According to the WHO, an estimated 17.9 million deaths occur annually due to CVDs, representing 31% of all global deaths.^(2)^ Among the various risk factors associated with CVDs, obesity is a significant concern, given its increasing and direct association with heart diseases.^(3)^ Body weight management is critical for individuals with or at risk of CVDs.^(4)^

Nutritional intervention is fundamental for managing obesity and consequently improving cardiovascular risk.^(5-7)^ It encompasses various approaches, including dietary counseling, personalized meal planning, and nutritional supplements, aimed at promoting lifestyle changes, healthier food choices, and maintaining a healthy weight.^(6)^ The potential benefits of nutritional intervention extend beyond weight loss and include improvements in various cardiovascular parameters crucial for individuals with high cardiovascular risk.^(7)^

One of the less explored areas of research is the impact of weight loss through nutritional intervention on the components of heart rate variability (HRV) in patients at high cardiovascular risk. Heart rate variability is a reliable indicator of autonomic nervous system function and cardiovascular health. The components of HRV provide valuable insights into sympathetic and parasympathetic activities. Understanding the effect of weight loss on these parameters could have significant implications for the management of CVDs. Moreover, patients at high cardiovascular risk often require a multi-drug regimen to manage their condition and prevent adverse cardiovascular events. The interplay between nutritional intervention, weight loss, and multi-drug regimens influencing HRV remains to be fully elucidated.

## OBJECTIVE

This study investigated the significant impact of moderate weight on the components of heart rate variability and biochemical profiles in obese patients with high cardiovascular risk through an institutional nutritional program.

## METHODS

### Study population and procedures

This was a non-randomized, uncontrolled study designed to evaluate the effectiveness of an institutional nutritional program. The study was conducted between November 2020 and July 2021 at the *Instituto Dante Pazzanese de Cardiologia*, São Paulo, Brazil. It is a tertiary referral center for cardiology, encompassing all subspecialties and offering comprehensive medical services. Under the supervision of a nutritionist and cardiologist, 36 patients with obesity (body mass index [BMI] >30kg/m²), aged 42-80 years and of either sex, were screened. The exclusion criteria were malignant disease, chronic kidney disease, heart failure, atrial fibrillation, hepatic disease, pacemaker use, and right and/or left bundle branch block. The nutritional program was based on personal dietary and health-related quality-of-life recommendations. After 3 months of the nutritional program, 12 patients were excluded due to irregular attendance during the follow-up period or failure to attend the HRV evaluation. Therefore, 24 eligible patients were included in the final analysis. Biochemical data, comorbidities, and pharmacological therapy data were obtained from the medical records. The patients were advised to follow a low-fat and salt hypocaloric diet containing 1,500-1,900 kcal. They were encouraged to consume at least five portions of fruits and/or vegetables and two portions of legumes, fish, lean meat, and whole-grain foods daily. Moreover, they were monitored for 3 months by the same nutritionist. They attended monthly consultations for anthropometric evaluations and dietary adherence monitoring. The biochemical data were based on medical records obtained up to a maximum of 6 months before the baseline and post-nutritional program periods.

Heart rate variability measurements were performed in the morning (8-11 AM) to avoid hemodynamic or circadian effects. Recordings were obtained with participants in the supine position. Each session lasted 10 min, during which the initial 5 min were excluded to allow stabilization, and the subsequent 5 min were analyzed. The participants were instructed to remain quiet and breathe spontaneously. A six-channel digital electrocardiograph (ECG-PC TEB-MAV, MG, Brazil) was used. Heart rate variability was assessed using time domain and frequency components. The mean heart rate, standard deviation of all R-R intervals (SDNN, ms), root mean square of successive differences (rMSSD, ms), percentage of adjacent RR intervals with a difference in duration greater than 50ms (pNN50, %), triangular index, low-frequency power (LF, n.u), high-frequency power (HF, n.u), and absolute ratio (LF/HF) were obtained.

This study was approved by the local ethics committee of *Instituto Dante Pazzanese de Cardiologia* (CAAE: 38040920.7.0000.5462; #4.388.707). All patients provided written informed consent.

### Statistical analysis

All analyses were performed using SPSS version 21.0 for Windows (IBM Corporation, USA), and the level of significance was set at p<0.05. Descriptive statistics for patient characteristics were expressed as the mean ± standard error (SE) or as percentages. Categorical variables were compared using the χ^2^ test. Numerical variables were measured twice in the same patient at two times (baseline and post-nutritional program), and generalized estimating equations (GEEs) were used. These models can use any distribution from the non-linear exponential family to represent the response variables, and not just those with a normal distribution. Generalized estimating equations were applied to analyze the data after adjusting for independent variables (*e.g*., sex, age category, comorbidities, previous CV events, and medications). The correlation coefficients were determined and tested for significance using Pearson's regression. Delta differences between baseline and post-nutritional programs were calculated.

## RESULTS

In this study, we evaluated 24 obese patients at high cardiovascular risk with an average age of 62 years (range: 42-80 years, SD, 10 years). Of these, 45.8% were adults (five men and six women), and 54.2% were older (seven men and six women). In terms of racial distribution, 75% of the participants identified as white, whereas 25% identified as black or brown. Notably, 87.5% of the patients had hypertension, 50% had type 2 diabetes, and all presented with dyslipidemia. Furthermore, 66.7% reported a history of acute myocardial infarction (AMI), and 8.3% had both AMI and stroke. Most (95.8%) patients were non-smokers. Moreover, 87.5% of patients were on chronic and concurrent treatment with drugs targeting the renin-angiotensin system (RAS), β-blockers, and statins (Table 1). Throughout the study period, the therapeutic regimens remained unchanged. No COVID-19 cases were detected during the study period.

**Table 1:** General characteristics of the study population

Variables		
Age (years)		62.1 ± 2.05
Age category, n (%)		
	Adults	11 (45.8)
	Older	13 (54.2)
Sex, n (%)		
	Male	12 (50.0)
	Female	12 (50.0)
Race, n (%)		
	White	18 (75.0)
	Black or Brown	06 (25.0)
Comorbidities and risk factors, n (%)		
	Hypertension	21 (87.5)
	Type 2 diabetes	12 (50.0)
	Dyslipidemias	24 (100)
	AMI	16 (66.7)
	AMI + stroke	02 (8.3)
	Smoking	01 (4.2)
Medications, n (%)		
	RAS-acting drugs	21 (87.5)
	ß-blockers	21 (87.5)
Calcium Channel Blockers		13 (52.2)
	ASA	16 (66.7)
	Diuretics	14 (58.3)
	Statins	21 (87.5)
	Ezetimibe	07 (29.2)
	Hypoglycemic drugs	12 (50.0)
	Insulin	06(25.0)
	Vasodilators	05(20.8)

Values are expressed as Mean ± Standard Error of the Mean or Percentage.

AMI: acute myocardial infarction; ASA: acetylsalicylic acid; RAS: Renin-Angiotensin System.

After the nutritional program, the patients showed a significant reduction in body weight (*p* = 0.043) (Table 2). The mean percentage weight loss was 5.4% (range: 2.2-13.6%). After the nutritional program, the mean BMI was 32.6Kg/m^2^ (range: 27.7-44.8kg/m^2^), a significant decrease from the baseline value of 34.8Kg/m^2^ (range: 30.0-47.4kg/m^2^) (Table 2). In addition, there was a significant reduction in the waist circumference, with a mean percentage of 4.8%, ranging from 1% to 14% (Table 2). Regarding the biochemical profile, the fasting blood glucose level remained unchanged after the nutritional program, whereas the glycated hemoglobin values decreased significantly in the model adjusted for the use of insulin and oral hypoglycemic agents (Table 2). The levels of total cholesterol, triglyceride, and LDLc also decreased after the nutritional program in the model adjusted for the use of statins and ezetimibe. However, the HDLc levels remained unchanged (Table 2).

**Table 2:** Anthropometric parameters, biochemical profile, and components of heart rate variability at baseline and after the intervention period

Variables		Baseline (n=24)	After the nutritional program (n=24)	p value
Body weight (Kg)		95.1 ± 2.11	88.0 ± 2.08	0.043
BMI (Kg/m^2^)		34.9 ± 0.74	32.6 ± 0.73	0.042
Waist circumference (cm)		106.7 ± 1.58	101.9 ± 1.57	0.031
Adjusted by insulin and hypoglycemic drugs				
	Glucose (mg/dl)	109.6 ± 5.86	100.1 ± 5.50	0.242
	Glycated hemoglobin (%)	6.6 ± 0.15	6.0 ± 0.14	0.015
Adjusted by statins and ezetimibe				
	Total cholesterol (mg/dl)	178.8 ± 6.42	149.2 ± 6.41	0.001
	LDLc (mg/dl)	95.1 ± 6.07	78.2 ± 5.93	0.047
	HDLc (mg/dl)	42.4 ± 1.76	42.6 ± 1.77	0.958
	Triglycerides (mg/dl)	204.1 ± 17.70	146.4 ± 17.64	0.021
Adjusted by RAS-acting drugs, p-blockers, presence of type 2 diabetes, and previous cardiovascular events (AMI and stroke)				
Time domain components				
	Heart Rate (bpm)	59 ± 2.13	57 ± 2.14	0.536
	SDNN (ms)	87.9 ± 14.7	74.8 ± 14.8	0.530
	pNN50(%)	18.8 ± 4.3	20.5 ± 4.4	0.783
	rMSSD (ms)	110.2 ± 20.3	92.1 ± 20.2	0.530
	Triangular Index	8.1 ± 0.77	9.2 ± 0.77	0.342
Frequency-domain components				
	LF(n.u)	38.8 ± 2.4	24.9 ± 2.2	<0.001
	HF (n.u)	47.8 ± 3.1	66.7 ± 3.4	<0.001
	Ratio LF/HF	0.95 ± 0.08	0.43 ± 0.09	<0.001

Values are expressed as Mean ± Standard Error of the Mean and were analyzed using general linear adjusted models.

BMI: Body Mass Index, AMI: acute myocardial infarction; RAS: Renin-Angiotensin System; SDNN: Standard Deviation of All Normal RR Intervals Recorded in a Time Interval; pNN50: Percentage of Adjacent RR Intervals with a Difference in Duration Greater than 50ms; rMSSD: Root Mean Square of Differences between Adjacent Normal RR Intervals in a Time Interval; LF: low frequency; HF: high frequency.

**Figure 1:**
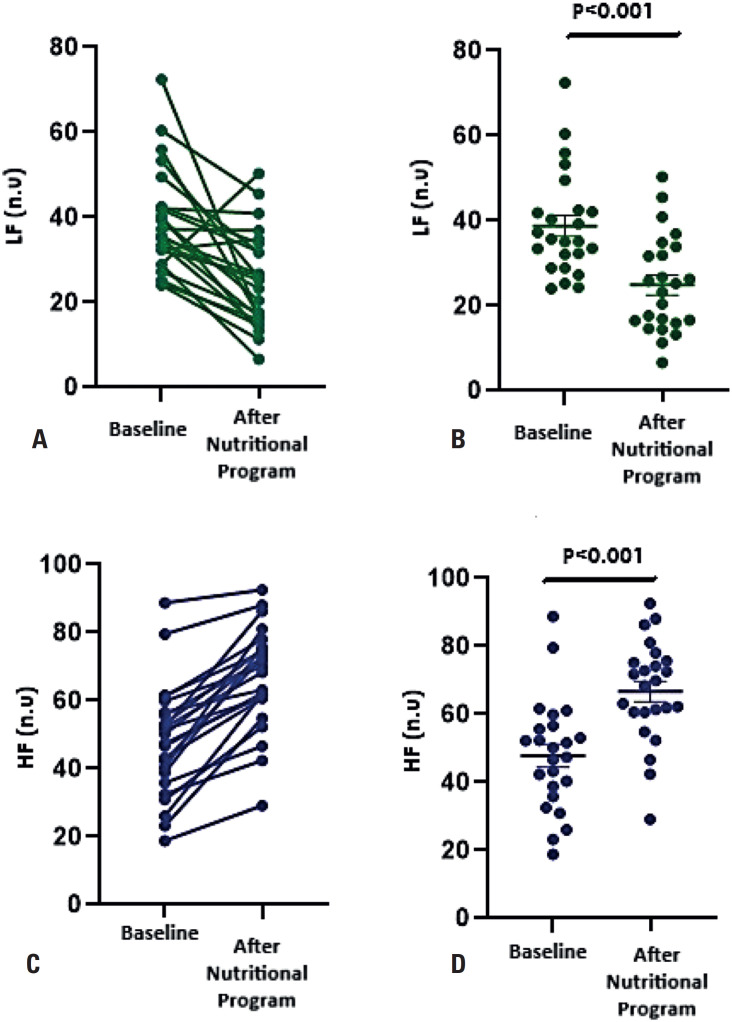
Effects of the nutritional program on the frequency-domain components in patients at high cardiovascular risk. In panels (A) and (B), the closed circles indicate individual values of LF at baseline and after the nutritional program. In panels (C) and (D), the closed circles indicate individual values of HF at baseline and after the nutritional program

**Figure 2:**
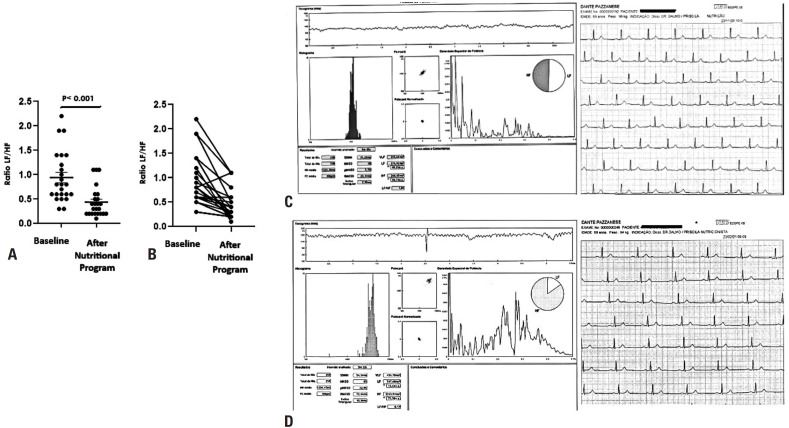
Effects of the nutritional program on the LF/HF ratio in patients at high cardiovascular risk. In panels (A) and (B), the closed circles indicate individual values of the LF/HF ratio at baseline and after the nutritional program. Panel (C) depicts a representative image of heart rate variability in the time (left) and frequency (right) domains, showing the SDNN, pNN50, and RMSSD components, as well as the LF and HF components, respectively. The circle on the right illustrates the balance between the high-frequency (HF, vagal activity) and low-frequency (LF, sympathetic activity) components at baseline, with an LF/HF ratio of 1.04. The electrocardiogram recording on the right shows only the D2 lead. Panel (D) presents a representative image of heart rate variability in the time (left) and frequency (right) domains, showing the SDNN, pNN50, and RMSSD components, as well as the LF and HF components, respectively. The circle on the right illustrates the increased significant difference in the high-frequency component (HF, vagal activity) in relation to the LF component (sympathetic activity) after the nutritional program, with an LF/HF ratio of 0.17. The electrocardiogram recording on the right shows only the D2 lead

The HRV components were evaluated in models adjusted for RAS-acting drugs, β-blockers, and previous cardiovascular events (AMI and stroke). No significant differences were observed between the baseline and post-nutritional program for heart rate (HR), SDNN, pNN50, rMSSD, or the triangular index (Table 2). However, the LF index significantly decreased after the nutritional program (Table 2) (Figure 1A, 1B). Significant effects of sex and age were observed. A greater reduction was observed among females (baseline: 42.3 ± 4.49; post-nutritional program: 25.4 ± 2.70; p=0.008) and adult patients (baseline: 37.9 ± 4.23; post-nutritional program: 22.3 ± 2.49; p=0.009) than among older and male patients. On the other hand, the HF index increased after the nutritional program when compared to the baseline period (p<0.001) (Table 2) (Figure 1C, 1D). This increase was more evident among males (baseline: 44.7 ± 3.79; post-nutritional program: 65.6 ± 5.56; p=0.011) and adult patients (baseline: 44.6 ± 3.91; post-nutritional program: 65.4 ± 5.73; p=0.017). The LF/HF ratio was lower after the nutritional program as compared to baseline (Table 2) (Figure 2). Moreover, no significant effects of sex and age were observed.

p<0.001 when comparing patients at baseline and after the nutritional program.

LF: low frequency; HF: high frequency.

p<0.001 when comparing patients at baseline and after the nutritional program.

LF: low frequency; Hf: high frequency.

After adjusting for sex, age category, RAS-acting drugs, β-blockers, and the presence of previous cardiovascular events (AMI and stroke), we found that the reduction in BMI was inversely correlated with the change in LF (r=0.471; p=0.042) and HF (r=0.231; p=0.047) indices.

## DISCUSSION

Several studies have revealed that adverse effects on HRV components tend to normalize after weight loss.^(8-10)^ However, reports on the impact of weight loss in patients at high cardiovascular risk remain limited.^(11-13)^ The current study highlights the significant effectiveness of a nutritional program in promoting moderate weight loss and improving HRV and biochemical profiles in obese patients at high cardiovascular risk in a tertiary care hospital. Over a 3-month period, the patients experienced moderate reductions in body weight, BMI, and waist circumference, as well as improvements in key biochemical markers. Heart rate variability analysis revealed enhancements in autonomic balance, characterized by decreased LF and LF/HF ratios, as well as increased HF after the nutritional program. These findings suggest that moderate weight loss can positively influence autonomic function and cardiovascular risk factors, offering a promising avenue for cardiovascular risk management in high-risk populations.

The nutritional program was effective for weight loss, characterized by a reduction in body weight, BMI, and waist circumference. Additionally, improvements in the biochemical profile, as evidenced by reductions in the levels of glycated hemoglobin, total cholesterol, triglycerides, and LDL-c, confirmed the effectiveness of this nutritional approach in individuals at high cardiovascular risk. In the current study, the mean weight reduction was 7.1 kg at the end of the 3-month intervention, representing a greater weight reduction than that reported by other studies that associated a hypocaloric diet with lifestyle changes.^(14)^ Moreover, low-calorie diets combined with lifestyle modification strategies were effective in promoting weight loss of between 5 and 8 kg over a period of 6 months, with a mean reduction in waist circumference of 4.2cm.^(15)^

Regarding the biochemical profile, a reduction in glycated hemoglobin levels was observed after the nutritional program. A meta-analysis that evaluated the positive effects of several intervention dietary protocols showed a significant reduction in glycated hemoglobin levels.^(16)^ Another nutritional intervention study found that after 6 months of follow-up, those who reached between 5-10% weight reduction at the end of the program achieved a significant improvement in glycated hemoglobin levels.^(17)^ Furthermore, a systematic review and meta-analysis of randomized clinical trials with nutritional intervention found a significant reduction in the concentrations of both total cholesterol and LDL-c, predicting a reduction of approximately 13% in the Framingham risk score for CVD over 10 years.^(18)^ Another systematic review revealed the positive effects of nutritional programs on reducing LDL-c levels.^(19)^ In this context, our findings are consistent with the observation that effective nutritional programs can reduce body composition and improve glycemic and lipid markers in patients at high cardiovascular risk. Therefore, these patients will have a new physiological state with a lower body weight and a better biochemical profile, promoting risk reduction attributable to several concomitant disorders.

In the current study, no significant differences were observed in the HR values or linear components after weight loss. These findings are in contrast with previous results reported in the literature.^(9,11,17,20,21)^ These discrepancies could be due to demographic differences and the clinical heterogeneity of the populations studied. Our study evaluated adults and older individuals of both sexes with several cardiometabolic complications treated with complex therapeutic regimens, some of whom had a history of AMI or stroke. Moreover, patients taking beta-blockers were not excluded from our analyses. Beta-blockers promote a reduction in sympathetic activity and increase the parasympathetic control of HR.^(9)^ It is conceivable that the weight loss observed in the current study did not produce a greater beneficial effect on HR and the linear components of HRV than that already occurring due to the chronic use of beta-blockers. Therefore, a long-term follow-up intervention study with more pronounced weight loss is necessary to confirm our findings in patients at high cardiovascular risk.

In contrast, there were significant changes in the frequency-domain components after weight loss due to the nutritional program. We observed a decrease in the LF index, which was more pronounced in adults and females. Furthermore, significant correlations were found between body weight, BMI changes, and LF index variation. The LF index, which is a derivative of the RR interval power spectrum, contains information on sympathetic and parasympathetic activities.^(22,23)^ Some authors have reported that weight loss after 16 weeks of nutritional intervention promoted a significant decline in the LF index among patients with obesity and type 2 diabetes.^(11)^ Another study also observed that weight reduction due to ketogenic and hypocaloric diet interventions was effective in reducing the LF index.^(24)^

The HF index, which is indicative of the vagal tone, increased after the nutritional program, especially in adult male patients. The greatest reductions in body weight and BMI significantly correlated with the largest increases in this index, suggesting that weight loss improves autonomic modulation through enhanced parasympathetic activity. Several studies have reported an increase in HF index after weight loss.^(9,21,24,25)^ Individuals undergoing intentional weight loss due to long-term calorie restriction showed an increase in HF index.^(9)^ Moreover, marked weight loss secondary to sleeve gastrectomy was effective in promoting a significant increase in the HF index.^(25)^

The LF/HF ratio, reflecting sympathovagal balance, significantly decreased after the nutritional program, consistent with literature findings that associate weight loss with parasympathetic predominance. A systematic review and meta-analysis concluded that weight loss was associated with a significant decrease in the LF/HF ratio, indicating parasympathetic predominance.^(26)^ Most studies in the literature have demonstrated that weight loss due to lifestyle changes appears to promote beneficial effects on HRV, restoring sympathetic-vagal balance, increasing parasympathetic activity, and reducing sympathetic activation.^(27)^ Despite known age and sex differences in HRV responses, our study did not observe an age effect on the frequency-domain components, possibly due to the specific demographics and comorbidities of our study population. However, post-nutritional program improvements in LF and HF indices were influenced by sex, with weight loss promoting a relative increase in vagal and parasympathetic activities in women and relative sympathetic dominance in men. These findings underscore the sex-specific effects of weight loss on HRV components and highlight the importance of considering sexual dimorphism in HRV analysis.

## CONCLUSION

Our findings suggest that the institutional nutrition program was effective in promoting moderate weight loss and improving autonomic function and cardiovascular risk factors, offering a promising avenue for cardiovascular risk management in high-risk populations undergoing multi-drug therapy in tertiary care centers. Despite these encouraging results, this study calls for further long-term research to explore the clinical implications.

## Data Availability

After publication, data will be available from the authors upon request—this condition is justified in the manuscript.
